# Supplementary material for: Positive and Negative Contribution from Lead–Oxygen Groups and Halogen Atoms to Birefringence: A First Principles Investigation
Source: Nanomaterials (Basel). 2023 Nov 28;13(23):3037. doi: 10.3390/nano13233037 (PMC10708153; doi:10.3390/nano13233037)
Supplement: Supplementary file 1 [file nanomaterials-13-03037-s001.zip › nanomaterials-2698183-supplementary.pdf]

Communication

Positive and Negative Contribution from Lead–Oxygen Groups and Halogen Atoms to Birefringence: A First Principles Investigation

Can Deng [1,†], Jialong Wang [1,†], Mei Hu [1], Xiuhua Cui [1,*], Haiming Duan [1], Peng Li [1,*] and Ming-Hsien Lee [2]

[1] Xinjiang Key Laboratory of Solid State Physics and Devices, School of Physical Science and Technology, Xinjiang University, 777 Huarui Street, Urumqi 830017, China; dc202407@163.com (D.C.); 107552200765@stu.xju.edu.cn (J.W.); humei09292022@163.com (M.H.); dhm@xju.edu.cn (H.D.)
[2] Department of Physics, Tamkang University, New Taipei City 25137, China; mhslee@mail.tku.edu.tw
* Correspondence: xjcxh0991@xju.edu.cn (X.C.); lip@xju.edu.cn (P.L.)
† These authors contributed equally to this work.

Table S1. The obtained parameters of $Pb_3O_2X_2$ unit cell.

Crystal		a	b	c	
$Pb_3O_2Cl_2$	measured	11.81 (Å)	5.78 (Å)	9.48 (Å)	α = 90.00° β = 90.00 ° γ = 90.00 °
	DFT-optimized	11.91 (Å)	5.87 (Å)	9.58 (Å)	
$Pb_3O_2Br_2$	measured	12.24 (Å)	5.87 (Å)	9.80 (Å)	α = 90.00° β = 90.00 ° γ = 90.00 °
	DFT-optimized	12.31 (Å)	5.93 (Å)	9.80 (Å)	
$Pb_3O_2Br_2$	measured	17.86 (Å)	5.95 (Å)	7.49 (Å)	α = 90.00° β = 90.00 ° γ = 90.00 °
	DFT-optimized	17.95 (Å)	5.98 (Å)	7.55 (Å)	

Table S2. The obtained parameters of $Pb_3O_2Cl_2$ atomic coordinates.

Atom	X	Y	Z
Pb1	0.4173156502772774	0.2500000000000000	0.4199853281517627
Pb2	0.0826843497227226	0.7500000000000000	0.9199853281517626
Pb3	0.5826843497227225	0.7500000000000000	0.5800146718482374
Pb4	0.9173156502772775	0.2500000000000000	0.0800146718482373
Pb5	0.7177606125402565	0.2500000000000000	0.3625412641039831
Pb6	0.7822393874597435	0.7500000000000000	0.8625412641039827
Pb7	0.2822393874597435	0.7500000000000000	0.6374587358960173
Pb8	0.2177606125402565	0.2500000000000000	0.1374587358960170
Pb9	0.4403624911090035	0.2500000000000000	0.8007650891486422
Pb10	0.0596375088909964	0.7500000000000000	0.3007650891486421
Pb11	0.5596375088909965	0.7500000000000000	0.1992349108513579
Pb12	0.9403624911090035	0.2500000000000000	0.6992349108513578
O1	0.5784517083430861	0.9953405670090483	0.3838724514552975
O2	0.9215482916569139	0.4953405670090480	0.8838724514552977
O3	0.9215482916569139	0.0046594329909519	0.8838724514552977
O4	0.4215482916569139	0.4953405670090480	0.6161275485447023
O5	0.4215482916569139	0.0046594329909519	0.6161275485447023
O6	0.0784517083430861	0.5046594329909517	0.1161275485447024
O7	0.0784517083430861	0.9953405670090483	0.1161275485447024
O8	0.5784517083430861	0.5046594329909517	0.3838724514552975
Cl1	0.6919474312753680	0.2500000000000000	0.6892437420777858
Cl2	0.8080525687246320	0.7500000000000000	0.1892437420777857
Cl3	0.3080525687246320	0.7500000000000000	0.3107562579222143
Cl4	0.1919474312753681	0.2500000000000000	0.8107562579222142
Cl5	0.6325238645129688	0.2500000000000000	0.0766019771799739
Cl6	0.8674761354870312	0.7500000000000000	0.5766019771799735
Cl7	0.3674761354870313	0.7500000000000000	0.9233980228200265
Cl8	0.1325238645129686	0.2500000000000000	0.4233980228200260

Table S3. The obtained parameters of $Pb_3O_2Br_2$ atomic coordinates.

Atom	X	Y	Z
Pb1	0.4202179399913980	0.2500000000000000	0.4236458691973263
Pb2	0.0797820600086021	0.7500000000000000	0.9236458691973263
Pb3	0.5797820600086023	0.7500000000000000	0.5763541308026737
Pb4	0.9202179399913977	0.2500000000000000	0.0763541308026738
Pb5	0.7114980490723395	0.2500000000000000	0.3629744891064018
Pb6	0.7885019509276605	0.7500000000000000	0.8629744891064016
Pb7	0.2885019509276606	0.7500000000000000	0.6370255108935984
Pb8	0.2114980490723394	0.2500000000000000	0.1370255108935983
Pb9	0.4433200753819853	0.2500000000000000	0.7922096347158220
Pb10	0.0566799246180148	0.7500000000000000	0.2922096347158223
Pb11	0.5566799246180149	0.7500000000000000	0.2077903652841779
Pb12	0.9433200753819851	0.2500000000000000	0.7077903652841780
O1	0.5763138769544298	0.9964180328279307	0.3864718997087028
O2	0.9236861230455702	0.4964180328279306	0.8864718997087031
O3	0.9236861230455702	0.0035819671720693	0.8864718997087031
O4	0.4236861230455703	0.4964180328279306	0.6135281002912969
O5	0.4236861230455703	0.0035819671720693	0.6135281002912969
O6	0.0763138769544297	0.5035819671720693	0.1135281002912972
O7	0.0763138769544297	0.9964180328279307	0.1135281002912972
O8	0.5763138769544298	0.5035819671720693	0.3864718997087028
Br1	0.6902822106419052	0.2500000000000000	0.6938209252107238
Br2	0.8097177893580948	0.7500000000000000	0.1938209252107237
Br3	0.3097177893580951	0.7500000000000000	0.3061790747892763
Br4	0.1902822106419050	0.2500000000000000	0.8061790747892762
Br5	0.6294465551676828	0.2500000000000000	0.0713850082791665
Br6	0.8705534448323172	0.7500000000000000	0.5713850082791665
Br7	0.3705534448323172	0.7500000000000000	0.9286149917208335
Br8	0.1294465551676828	0.2500000000000000	0.4286149917208334

Table S4. The obtained parameters of $Pb_3O_2I_2$ atomic coordinates.

Atom	X	Y	Z
Pb1	0.0279754050345063	0.2500000000000000	0.1505510721806704
Pb2	0.4720245949654938	0.7500000000000000	0.6505510721806704
Pb3	0.9720245949654936	0.7500000000000000	0.8494489278193296
Pb4	0.5279754050345064	0.2500000000000000	0.3494489278193295
Pb5	0.3380544331895737	0.2500000000000000	0.5261585322581450
Pb6	0.1619455668104262	0.7500000000000000	0.0261585322581448
Pb7	0.6619455668104262	0.7500000000000000	0.4738414677418551
Pb8	0.8380544331895738	0.2500000000000000	0.9738414677418550
Pb9	0.6073308692510272	0.2500000000000000	0.7921873739057215
Pb10	0.8926691307489728	0.7500000000000000	0.2921873739057216
Pb11	0.3926691307489729	0.7500000000000000	0.2078126260942784
Pb12	0.1073308692510272	0.2500000000000000	0.7078126260942785
I1	0.2411181127595374	0.2500000000000000	0.1716851851803514
I2	0.2588818872404626	0.7500000000000000	0.6716851851803514
I3	0.7588818872404627	0.7500000000000000	0.8283148148196486
I4	0.7411181127595373	0.2500000000000000	0.3283148148196487
I5	0.4347135857050545	0.2500000000000000	0.9083721835325103
I6	0.0652864142949454	0.7500000000000000	0.4083721835325101
I7	0.5652864142949452	0.7500000000000000	0.0916278164674898
I8	0.9347135857050548	0.2500000000000000	0.5916278164674897
O1	0.4248982250069957	0.0001581549454682	0.4177248966428499
O2	0.0751017749930043	0.5001581549454681	0.9177248966428500
O3	0.0751017749930043	-0.0001581549454681	0.9177248966428500
O4	0.5751017749930045	0.5001581549454681	0.5822751033571500
O5	0.5751017749930045	-0.0001581549454681	0.5822751033571500
O6	0.9248982250069955	0.4998418450545317	0.0822751033571502
O7	0.9248982250069955	0.0001581549454682	0.0822751033571502
O8	0.4248982250069957	0.4998418450545317	0.4177248966428499

Table S5. The obtained static dielectric matrix.

Crystal	X	Y	Z
	5.10792	0	0
$Pb_3O_2Cl_2$	0	5.122361	0
	0	0	4.996154
	5.697997	0	0
$Pb_3O_2Br_2$	0	5.623431	0
	0	0	5.453133
	6.322345	0	0
$Pb_3O_2I_2$	0	6.448983	0
	0	0	6.171130

Table S6. The obtained Born effective charges of $Pb_3O_2Cl_2$.

Crystal	atom	q_{xx}	q_{yy}	q_{zz}
		-2.11584	-0.04772	0.22352
	O1	-0.41283	-3.28532	-0.13238
		0.39632	0.10131	-2.27909
		-2.11584	0.04772	-0.22352
	O2	0.41283	-3.28532	-0.13238
		-0.39632	0.10131	-2.27909
		-2.11584	-0.04772	-0.22352
	O3	-0.41283	-3.28532	0.13238
		-0.39632	-0.10131	-2.27909
		-2.11584	0.04772	0.22352
	O4	0.41283	-3.28532	0.13238
		0.39632	-0.10131	-2.27909
		-2.53999	0	-0.0095
	Cl1	0	-1.61943	0
		-0.15136	0	-1.6015
		-2.53999	0	0.0095
	Cl2	0	-1.61943	0
		0.15136	0	-1.6015
		-1.49253	0	-0.169
	Cl3	0	-1.75796	0
		-0.25634	0	-2.03922
$Pb_3O_2Cl_2$		-1.49253	0	0.169
	Cl4	0	-1.75796	0
		0.25634	0	-2.03922
		2.89843	0	-0.2662
	Pb1	0	3.54836	0
		-0.21271	0	2.46643
		2.89843	0	0.2662
	Pb2	0	3.54836	0
		0.21271		2.46643
		2.56882	0	-0.15219
	Pb3	0	3.12347	0
		-0.47045	0	2.75109
		2.56882	0	0.15219
	Pb4	0	3.12347	0
		0.47045	0	2.75109
		2.79695	0	-0.25707
	Pb5	0	3.27619	0
		-0.18067	0	2.98138
		2.79695	0	0.25707
	Pb6	0	3.27619	0
		0.18067	0	2.98138

Table S7. The obtained Born effective charges of $Pb_3O_2Br_2$.

Crystal	atom	q_{xx}	q_{yy}	q_{zz}
		-2.14091	-0.07709	0.24095
	O1	-0.4672	-3.4366	-0.13938
		0.42822	0.03023	-2.39448
		-2.14091	0.07709	-0.24095
	O2	0.4672	-3.4366	-0.13938
		-0.42822	0.03023	-2.39448
		-2.14091	-0.07709	-0.24095
	O3	-0.4672	-3.4366	0.13938
		-0.42822	-0.03023	-2.39448
		-2.14091	0.07709	0.24095
	O4	0.4672	-3.4366	0.13938
		0.42822	-0.03023	-2.39448
		-2.80378	0	-0.00424
	Br1	0	-1.62696	0
		-0.10324	0	-1.60854
		-2.80378	0	0.00424
	Br2	0	-1.62696	0
		0.10324	0	-1.60854
		-1.40402	0	-0.15908
	Br3	0	-1.79283	0
		-0.30164	0	-2.14664
**$Pb_3O_2Br_2$**		-1.40402	0	0.15908
	Br4	0	-1.79283	0
		0.30164	0	-2.14664
		2.8812	0	-0.21835
	Pb1	0	3.68426	0
		-0.25242	0	2.50932
		2.8812	0	0.21835
	Pb2	0	3.68426	0
		0.25242	0	2.50932
		2.50052	0	-0.13904
	Pb3	0	3.27389	0
		-0.37698	0	3.00411
		2.50052	0	0.13904
	Pb4	0	3.27389	0
		0.37698	0	3.00411
		3.10697	0	-0.31639
	Pb5	0	3.33485	0
		-0.19102	0	3.03071
		3.10697	0	0.31639
	Pb6	0	3.33485	0
		0.19102	0	3.03071

Table S8. The obtained Born effective charges of $Pb_3O_2I_2$.

Crystal	atom	q_{xx}	q_{yy}	q_{zz}
		-2.50878	0.29931	0.22778
	O1	0.1664	-3.6608	-0.61266
		0.11855	-0.23359	-2.55461
		-2.50878	-0.29931	-0.22778
**$Pb_3O_2I_2$**	O2	-0.1664	-3.6608	-0.61266
		-0.11855	-0.23359	-2.55461
		-2.50878	0.29931	-0.22778
	O3	0.1664	-3.6608	0.61266
		-0.11855	0.23359	-2.55461

	O4	-2.50878	-0.29931	0.22778
		-0.1664	-3.6608	0.61266
		0.11855	0.23359	-2.55461
	I1	-2.07598	0	-1.04704
		0	-1.81465	0
		-0.86623	0	-1385534
	I2	-2.07598	0	1.04704
		0	-1.81465	0
		0.86623	0	-1385534
	I3	-1.77951	0	-0.37329
		0	-1.56772	0
		-0.17454	0	-2.04128
	I4	-1.77951	0	0.37329
		0	-1.56772	0
		0.17454	0	-2.04128
	I1	2.99927	0	-0.41559
		0	3.67755	0
		-0.18939	0	2.76695
	Pb2	2.99927	0	0.41559
		0	3.67755	0
		0.18939	0	2.76695
	Pb3	3.65701	0	0.17498
		0	3.45576	0
		-0.04174	0	2.98908
	Pb4	3.65701	0	-0.17498
		0	3.45576	0
		0.04174	0	2.98908
	Pb5	2.21677	0	-0.22123
		0	3.57065	0
		0.10294	0	3.24982
	Pb6	2.21677	0	0.22123
		0	3.57065	0
		-0.10294	0	3.24982

Table S9. The obtained diagonal elements and the difference of Born effective charges.

Crystal	atom	q_{xx}	q_{yy}	q_{zz}	Δq
**$Pb_3O_2Cl_2$**	Cl1	-2.53999	-1.61943	-1.60150	-0.0179
	Cl2	-1.49253	-1.75796	-2.03922	0.28126
	Pb1	2.89843	3.54836	2.46643	1.08193
	Pb2	2.56882	3.12347	2.75109	0.37238
	Pb3	2.79695	3.27619	2.95138	0.29481
**$Pb_3O_2Br_2$**	Br1	-2.80378	-1.62696	-1.60854	-1.19524
	Br2	-1.40402	-1.79283	-2.14664	0.74262
	Pb1	2.88212	3.68426	2.50932	0.37280
	Pb2	2.50052	3.27389	3.00411	-0.50359
	Pb3	3.10697	3.33485	3.03071	0.07626
**$Pb_3O_2I_2$**	I1	-2.07598	-1.81465	-1.85534	0.04069
	I2	-1.77951	-1.56772	-2.04128	0.47356
	Pb1	2.99927	3.67755	2.76695	0.91060
	Pb2	3.65701	3.45576	2.98908	0.46668
	Pb3	2.21677	3.57065	3.24982	0.32083

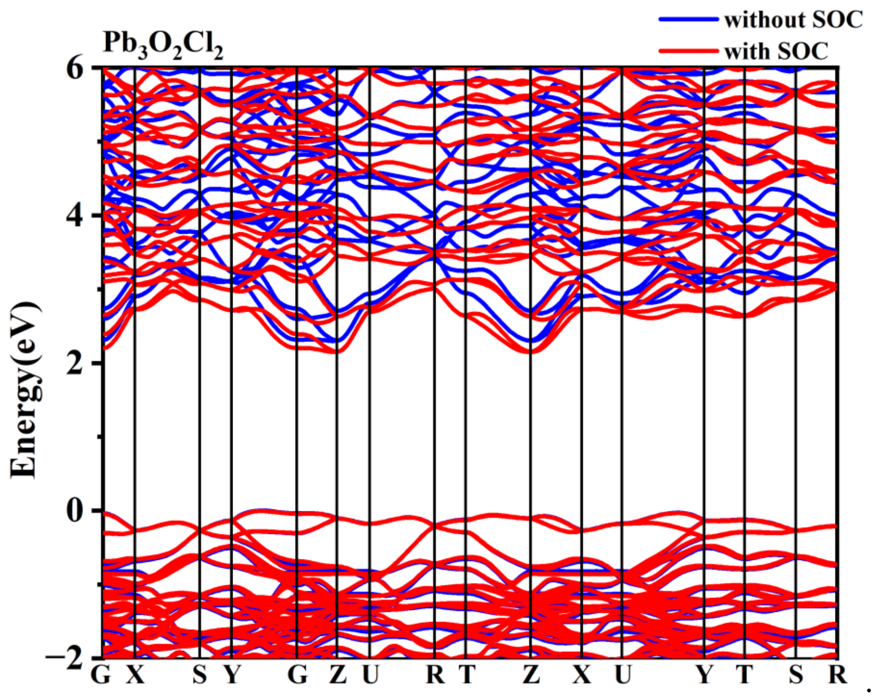

Figure S1. The obtained band structures with and without concerning SOC.
